# Comparative Assessment of Clinical Outcomes in Flapless and Flapped Implant Surgical Techniques: A Prospective Cohort Study

**DOI:** 10.7759/cureus.82547

**Published:** 2025-04-18

**Authors:** Kenil J Tarpara, Rohit Goyal, Niyutna Sheshamani, Navneet Singh, Hem Kumar, Waseem F Mirdah, Seema Gupta

**Affiliations:** 1 Department of Oral and Maxillofacial Surgery, Maharaja Ganga Singh Dental College and Research Centre, Sri Ganganagar, IND; 2 Department of Orthodontics, Kothiwal Dental College and Research Centre, Moradabad, IND

**Keywords:** dental implants, flap, flapless, post-operative pain, probing

## Abstract

Introduction: Dental implants have become the standard treatment for restoring missing teeth, offering both functional and esthetic benefits. The surgical approach (flap versus flapless) may influence peri-implant tissue healing, postoperative discomfort, and long-term implant success. This study aimed to compare postoperative pain, peri-implant probing depth (PD), and crestal bone height (CBH) changes between the flapless and flapped implant placement over a 12-month period post functional loading of implants.

Materials and methods: A prospective cohort study was conducted with 20 patients who required single posterior dental implants. Patients were allocated into two groups: flapless (n = 10) and flap (n = 10). The allocation was based on clinical evaluation and patient preferences. Soft tissue assessment included PD measurements at six and 12 months at the four peri-implant sites using a periodontal probe. Hard tissue assessment included CBH measurements at baseline, six months, and 12 months post functional loading using standardized intraoral radiographs. Pain intensity was evaluated on postoperative days 3 and 7 using the visual analog scale (VAS). All data were analyzed using appropriate parametric and non-parametric tests, with significance set at p < 0.05.

Results: Postoperative pain was significantly lower in the flapless group compared to the flap group at both day 3 (3.1 ± 0.74 vs. 5.7 ± 0.95, p = 0.001) and day 7 (0.5 ± 0.71 vs. 2.4 ± 1.17, p = 0.001). PD was significantly lower in the flapless group at six months (p < 0.05), although by 12 months, the differences between the groups were not statistically significant. Both groups showed significant intragroup reductions in PD over time (p = 0.001). The CBH decreased slightly in both groups over 12 months, but no significant intergroup differences were observed at any time point (p > 0.05).

Conclusion: Flapless implant placement was associated with significantly lower postoperative pain and more favorable soft tissue healing with reduced PD at six months than the flap technique. However, both approaches demonstrated comparable changes in CBH at 12 months. These findings suggest that flapless surgery may offer early postoperative benefits without compromising long-term hard tissue stability.

## Introduction

The primary goal of modern dentistry is to restore patients to a state of normal contour, function, comfort, esthetics, speech, and overall oral health by addressing diseases or by replacing lost teeth. Among the various restorative options, dental implantology stands out because of its ability to achieve these objectives regardless of any pathological or traumatic damage to the stomatognathic system [1].

Dental implants are biocompatible, alloplastic materials that are surgically inserted into the jawbone (endosseous) or on the bone surface (subperiosteal) to provide support for fixed or removable prosthetic restorations [2]. Implant-supported prostheses have become a highly effective and predictable method for the replacement of missing teeth, with long-term success rates ranging between 90% and 100% over a 10-year period [3]. Their popularity stems from their ability to restore both esthetics and function in patients who have lost teeth due to disease, trauma, or other causes. Titanium and its alloys are commonly used in dental implants because of their biocompatibility and inertness, which reduce the risk of adverse biological reactions [2].

The key to the success of dental implants is osseointegration, a direct and functional connection between the implant and surrounding living bone, free of any intervening soft tissue [2]. However, contemporary implantology now emphasizes the critical role of peri-implant soft tissues, as their health significantly affects the long-term success and esthetic outcomes of implants. The preservation of both soft tissues, such as the gingiva, and hard tissues, particularly the crestal bone, is essential for maintaining implant stability and preventing complications such as peri-implantitis [4].

Among the surgical techniques available for implant placement, two predominant approaches are recognized: the conventional flap technique and flapless technique. The conventional method involves elevation of a full-thickness mucoperiosteal flap to expose the bone, whereas the flapless technique places the implant without raising a flap, often using a tissue punch or guided surgery. The results of both techniques are controversial. Some studies suggest that the flapless technique results in superior preservation of soft tissue architecture and crestal bone levels owing to reduced surgical trauma and better maintenance of blood supply to the periosteum [5,6]. These studies reported lower postoperative morbidity, faster healing, and less crestal bone resorption with flapless procedures. Conversely, other studies have highlighted the benefits of the flap technique, particularly in providing direct visualization of the bone and surrounding anatomical structures, which allows for more precise implant positioning and bone contouring when required [7,8]. Moreover, some studies indicated no significant difference between the two techniques in terms of long-term implant survival and crestal bone loss, suggesting that both techniques can be equally effective when properly planned and executed [9,10]. Given the importance of preserving both soft and hard tissues for longevity and success of dental implants, it is crucial to evaluate the influence of these two surgical techniques on peri-implant tissue stability. Therefore, the present study aimed to comparatively assess soft and hard tissue changes following endosseous implant placement using flap and flapless techniques over a 12-month period post-functional loading of implants, thereby contributing valuable insights into optimal clinical practices for implant therapy.

## Materials and methods

Study design and setting

This prospective cohort study was conducted at the Department of Oral and Maxillofacial Surgery, Maharaja Ganga Singh Dental College and Research Center, Sri Ganganagar, Rajasthan. The study observed naturally occurring groups of patients who required a single posterior endosseous dental implant. Over a period of two years (January 2023 to January 2025), patients were prospectively followed after being allocated into two groups (flap vs. flapless) based on clinical judgment, operator preference, and patient-specific factors (e.g., soft tissue biotype, bone quantity, and quality). No randomization was applied, and groups were formed based on real-world clinical decision-making, reflective of how implant placement is typically performed in everyday clinical practice. The study was approved by the Institutional Ethics Committee (MGSDC|SY/23/05) and followed the principles of the Declaration of Helsinki. Written informed consent was obtained from all patients before starting the study. 

Patients' eligibility 

Patients presenting to the department were screened according to pre-established inclusion and exclusion criteria. Only patients with a single missing posterior tooth were included in the study to standardize surgical procedures and reduce variability in outcome measures. Inclusion criteria were adults aged 18 to 60 years, requiring a single posterior endosseous dental implant, with sufficient alveolar ridge dimensions (minimum buccolingual width of 6 mm, vertical bone height sufficient for an implant of at least 10 mm), minimum interocclusal clearance of 7 mm, good oral hygiene (plaque and bleeding index of 0 or 1), and adequate keratinized mucosa at the implant site. Bone quality and quantity were assessed clinically and radiographically prior to inclusion. The presence of adequate keratinized mucosa at the implant site was confirmed clinically by measuring the width of keratinized tissue using a UNC-15 periodontal probe (Hu-Friedy, Chicago, Illinois, USA). A minimum of 2 mm of keratinized mucosa in the buccolingual direction was considered sufficient for implant placement. Exclusion criteria included patients with systemic diseases (e.g., uncontrolled diabetes, osteoporosis or other metabolic bone disorders, bleeding disorders or those undergoing anticoagulant therapy, and autoimmune diseases such as rheumatoid arthritis and systemic lupus erythematosus, immunocompromised states, including HIV/AIDS and organ transplant recipients, chronic kidney or liver disease, cardiovascular conditions), any history of radiation therapy to the head and neck region, active periodontal infections, pregnancy or lactation, parafunctional habits such as severe bruxism, use of medications that could impair healing (e.g., bisphosphonates), smokers or users of smokeless tobacco, and those with multiple missing teeth or who required more than one implant. Only compliant and motivated patients willing to follow the study protocol and attend all follow-up visits were enrolled.

Sample size estimation

The sample size for this study was determined using G*Power software version 3.2.9 (Heinrich-Heine-Universität Düsseldorf, Düsseldorf, Germany) with a statistical power of 80% and an alpha error of 5%. An effect size of 0.85 was adopted, based on findings from a prior study [11]. This study utilized the crestal bone-level parameter after implant placement. The calculation yielded an estimated sample size of ten patients per group.

Methodology

Prior to implant placement, all patients underwent a thorough clinical and radiographic assessment of the implant site. Alveolar ridge measurements were considered sufficient if the buccolingual width was ≥ 6 mm and the vertical bone height was ≥ 10 mm. The buccolingual ridge width was measured clinically using a ridge mapping technique under local anesthesia. This involved marking the soft tissue at the implant site and using a periodontal probe or ridge-mapping caliper to palpate the underlying bone and determine the width at the crest. The vertical bone height was evaluated using standardized intraoral periapical radiographs (IOPAs) obtained with the paralleling technique and a custom radiographic stent incorporating a radiopaque marker for consistent image reproduction. Although cone-beam computed tomography (CBCT) provides superior three-dimensional evaluation, it was not employed in this study due to practical limitations and in an effort to reflect routine clinical protocols in resource-constrained settings. This approach, combining clinical measurements and reproducible 2D radiography, ensured sufficient site evaluation for implant placement in all included cases.

Baseline diagnostic records, including IOPA, radiovisiography (RVG), study models and clinical photographs were obtained. All radiographs were obtained by the same clinician with more than five years of experience. Patients were not randomly assigned to either the flap or the flapless group. Instead, allocation was performed according to the clinical scenario, patient-specific factors (such as soft tissue biotype), and surgeon’s clinical judgment, in conjunction with patient preference. Key considerations included gingival biotype, keratinized tissue width, ridge contour, soft tissue thickness, and anticipated ease of surgical access. Patients with thick biotypes, sufficient keratinized mucosa, and favorable ridge architecture were generally considered suitable for flapless implant placement, as these features supported soft tissue preservation and predictable healing. On the other hand, patients with thin biotypes, reduced tissue volume, or need for better surgical visualization were more likely to undergo flap elevation, enabling precise implant placement and soft tissue management. This individualized approach reflects real-world clinical decision-making rather than a randomized allocation model. Presurgical preparation involved administration of a prophylactic dose of amoxicillin (500 mg)- potassium clavulanate (125 mg) one hour before surgery, to reduce the risk of surgical site infections and peri-implantitis, particularly in the absence of active infection at the implant site.

Surgical protocol

All implants in this study were placed following a delayed placement protocol, at least three months after tooth extraction, ensuring adequate healing of the extraction site and soft tissue closure prior to surgery. A consolidation period of three months was allowed for osseointegration before proceeding with the second-stage surgery and prosthetic rehabilitation. All implants were restored using a delayed loading protocol, with the final prosthesis delivered only after confirmation of successful osseointegration. No immediate or provisional restorations were provided during the healing phase to minimize loading and ensure favorable implant stability.

Flapless Group (Cohort 1, n = 10)

Following local anesthesia (2% lignocaine with 1:80000 epinephrine), a tissue punch was used to create an access point, and osteotomy was performed under saline irrigation at 800-1000 rpm. A dental implant (Adin Dental Implant Systems Ltd., Afula, Israel) was then inserted. A depth gauge was used intermittently to ascertain the requisite depth. Once the predetermined depth has been accomplished, the implant is affixed to a carrier and subsequently driven by a motor to its definitive position with deliberation. Precautions were taken to ensure that the implant collar was aligned with the crest of the pre-existing alveolar ridge. Based on the diameter of the implant, a healing collar measuring 2 mm in height was positioned over the implant platform.

Flap Group (Cohort 2, n = 10)

After reflecting on a full-thickness mucoperiosteal flap via a mid-crestal incision, osteotomy was performed similarly. A cover screw was placed over the implant before flap closure using 3-0 silk sutures. Postsurgical instructions were provided to both groups, and sutures were removed on the seventh postoperative day in the flap group. After surgery, patients in both groups were provided with standard postsurgical instructions, including recommendations for maintaining oral hygiene (with a prescribed chlorhexidine mouthwash), managing pain and swelling with NSAIDs and ice packs, and adhering to a soft diet for the first week. They were advised to avoid physical exertion, chewing hard foods, or irritating the surgical site. Flap group patients were scheduled for suture removal on the seventh postoperative day, while flapless group patients were given instructions for managing the healing collar. Follow-up visits were planned at one week, one month, six months, and twelve months to assess healing and implant stability.

Outcome assessment

**Figure 1:**
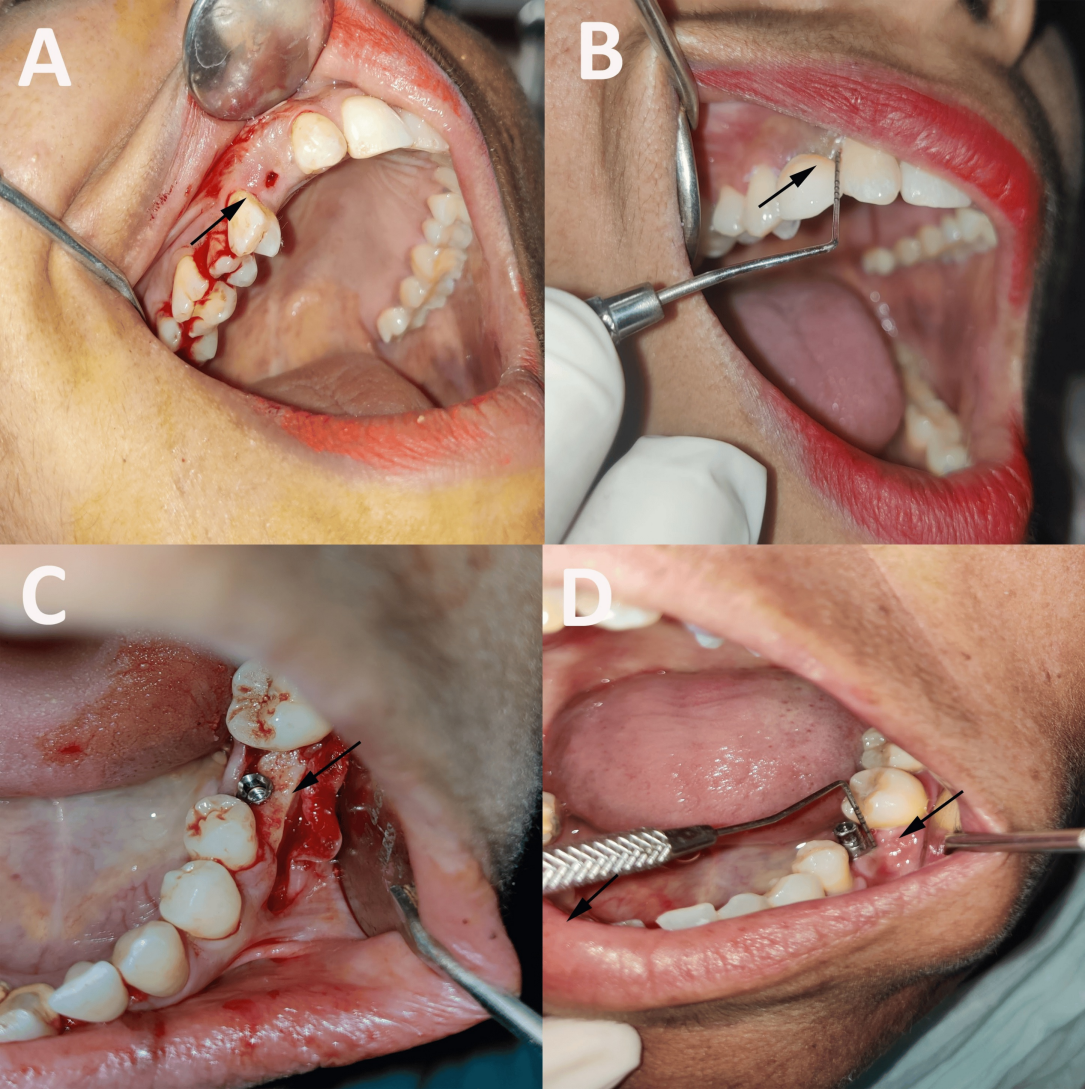
(A) Flapless implant placement, (B) Measuring probing depth around implant placed by the flapless procedure, (C) Flap surgery for implant placement, and (D) Measuring probing depth around implant placed by flap surgery. This figure represents images from patients who participated in this study. All identifying information has been removed to maintain confidentiality.

Soft tissue evaluation was performed, where the peri-implant probing depth (PD) was recorded at baseline, six months, and 12 months after functional loading to better assess long-term outcomes, using a plastic UNC-15 periodontal probe to avoid damaging the implant surface or peri-implant mucosa. Probing was performed at four sites around each implant: mesiobuccal, distobuccal, mesiolingual or mesiopalatal, and distolingual or distopalatal. Light probing force was maintained, and the mean values of the buccal and lingual aspects were used for analysis (Figure 1). The PD was measured around the functional restoration rather than the abutment at six- and 12-month post functional loading.

**Figure 2:**
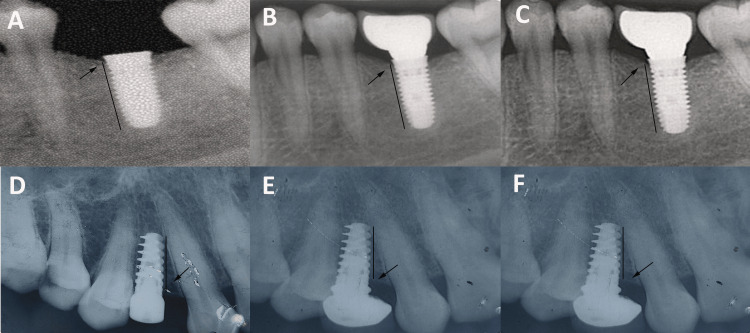
Crestal bone height: (A) at baseline in flapless surgery, (B) at the sixth month in flapless surgery, (C) at the 12th month in flapless surgery, (D) at baseline in flap surgery, (E) at the sixth month in flap surgery, and (F) at the 12th month in flap surgery. This figure represents radiographic images from patients who participated in this study. All identifying information has been removed to maintain confidentiality.

To evaluate peri-implant hard tissue changes, crestal bone height (CBH) measurements were performed on standardized IOPAs using a digital image analysis protocol. All radiographs were obtained using the paralleling technique with a Rinn XCP positioning device (Dentsply Sirona Inc., North Carolina, USA) and a custom-made bite-registration stent to ensure reproducibility at baseline, six months, and 12 months, after functional loading, along with a long cone technique (16-inch cone) to reduce magnification and distortion errors. The standardized IOPA films were either scanned using a high-resolution image scanner or directly captured as digital images via RVG, ensuring consistent exposure settings (kVp, mA, and exposure time) across all patients and time points. For each implant, the apex served as a fixed anatomical reference point for consistent measurements. The CBH was identified as the most coronal point of bone-implant contact (BIC) on both the mesial and distal aspects, where the alveolar bone meets the implant threads. The scanned images were analyzed using ImageJ Analysis Software (NIH, Bethesda, MD, USA), where each image was calibrated using the known implant length to convert the pixel values to mm. A straight line was drawn from the implant apex to the BIC on both the mesial and distal aspects, and the software automatically calculated linear distances in mm. The mesial and distal CBHs were recorded separately, and the mean of these two measurements was used to represent the mean CBH for each implant at all three time points (Figure 2).

Pain assessment was performed by recording pain intensity using the visual analog scale (VAS) on postoperative days 3 and 7. The VAS consisted of a 10-cm horizontal line, where the left end was marked as "0" representing no pain, and the right end was marked as "10" representing the worst imaginable or unbearable pain. The patients were asked to indicate a point along the line that best represented their perceived pain intensity on each postoperative day. The distance from "0" to the patient's mark was measured in cm and recorded as the pain score, providing a quantitative measure of postoperative discomfort at both time intervals. The patients were prospectively monitored over a 12-month follow-up period after functional loading of implants. Baseline data were collected using intraoral radiographs and clinical evaluation, and follow-up data were obtained at six months and 12 months post functional loading. Soft tissue outcomes (peri-implant PD) and hard tissue changes (CBH) were assessed at these time points. Pain levels were measured on postoperative days 3 and 7 using the VAS.

Blinding and reliability testing

All clinical and radiographic measurements were performed by a single, blinded, calibrated examiner to minimize inter-examiner variability. Intra-examiner reliability was verified by repeating 20% of the measurements at a one-week interval, and the intra-class correlation coefficient (ICC) was calculated.

Statistical analysis

Data were entered into Microsoft Excel (Microsoft Corporation, Redmond, USA) and analyzed using Stata Statistical Software Release 18 (StataCorp LLC, College Station, TX, USA). Categorical variables were expressed as frequencies and percentages, whereas continuous variables were described using means and standard deviations (SD). The Shapiro-Wilk test, supplemented by Q-Q plots, was used to check data normality. The VAS scores and periodontal depth were normally distributed, whereas CBH showed a non-normal distribution. For normally distributed data, intergroup comparisons were performed using the independent t-test and intragroup comparisons using the paired t-test. For non-normal data, the Mann-Whitney U test and Friedman test were applied. Statistical significance was established at a p-value of 0.05.

## Results

The ICC value of 0.85 showed good reliability and reproducibility. The flap group comprised six male patients (30%) and four female patients (20%), while the flapless group included seven male patients (35%) and three female patients (15%). Overall, the sample consisted of 13 male patients (65%) and seven female patients (35%). A chi-square test revealed no significant association between surgical technique and sex distribution (Table 1).

**Table 1:** Analysis of the association of sex with the surgical technique by the chi-square test. p-value > 0.05: non-significant. Data is presented in the form of frequency (n) and percentage (%).

Group	Total n (%)	Male n (%)	Female n (%)	Chi stat	p-value
Flap	10 (50%)	6 (30%)	4 (20%)	0.21	0.639
Flapless	10 (50%)	7 (35%)	3 (15%)		
Total	20 (100%)	13 (65%)	7 (35%)		

The descriptive statistics revealed that the mean age of patients was slightly higher in the flap group (37.4 ± 9.55 years) compared to the flapless group (36.5 ± 5.87 years). The implant length was nearly similar, with a mean of 11.4 ± 1.61 mm in the flap group vs. 11.25 ± 1.67 mm in the flapless group. However, the implant diameter was slightly larger in the flapless group than that in the flap group (Table 2).

**Table 2:** Descriptive statistics of study groups with implants placed with flap and flapless techniques. Data is presented in the form of frequency (n), percentage (%), mean, and standard deviation (SD).

Parameters	Group	n	%	Minimum	Maximum	Mean ± SD
Age (years)	Flap	10	50%	27	58	37.4 ± 9.55
	Flapless	10	50%	27	47	36.5 ± 5.87
Implant length (mm)	Flap	10	50%	10	15	11.4 ± 1.61
	Flapless	10	50%	10	15	11.25 ± 1.67
Implant diameter (mm)	Flap	10	50%	3.30	4.20	3.89 ± 0.30
	Flapless	10	50%	3.75	4.20	4.11 ± 0.19

The results indicated a significant difference in postoperative pain levels between the flap and flapless implant placement groups. On the third day, the mean VAS score was significantly higher in the flap group (5.7 ± 0.95) than in the flapless group (3.1 ± 0.74) (p = 0.001). By the seventh day, pain levels were reduced in both groups; however, the flap group (2.4 ± 1.17) still reported higher pain than the flapless group (0.5 ± 0.71) (p = 0.001). These findings suggest that flapless implant placement resulted in significantly lower postoperative pain and faster recovery than the conventional flap method (Table 3).

**Table 3:** Intergroup comparison using an independent t-test and intragroup comparison using a paired t-test for pain scores at various time intervals. *p-value < 0.05: significant. VAS: Visual analog scale. Data is presented in the form of mean and standard deviation (SD).

Time intervals	Flap	Flapless	t-stat	p-value (intergroup)
VAS at the third day	5.7 ± 0.95	3.1 ± 0.74	6.84	0.001*
VAS at the seventh day	2.4 ± 1.17	0.5 ± 0.71	4.38	0.001*
Paired t-test	15.46	8.51		
p-value (intragroup)	0.001*	0.001*		

The comparison of the PD at different time intervals between the flap and flapless implant placement groups revealed significant differences. At the sixth month, the mean PD values were significantly higher in the flap group in the mesiobuccal region (3.96 ± 0.57), mesiolingual (3.79 ± 0.49), distobuccal (3.86 ± 0.58), and distolingual (3.72 ± 0.51) sites compared to the flapless group (p < 0.05). However, at the 12th month, the PD values decreased in both groups, but the difference was not statistically significant (p > 0.05). Paired t-tests showed significant reductions in PD in both groups over time (p = 0.001). These results suggest that flapless implant placement was associated with a lower initial PD and a more favorable healing response than flap surgery, although both techniques showed improvement over time. These findings highlight the potential benefits of the flapless approach in preserving peri-implant soft tissues (Table 4).

**Table 4:** Intergroup comparison using an independent t-test and intragroup comparison using a paired t-test for the probing depth (PD) in mm at various time intervals. *p-value < 0.05: significant; MB: mesiobuccal, ML: mesiolingual, DB: distobuccal, DL: distolingual. Data is presented in the form of mean and standard deviation (SD).

Time intervals	Flap	Flapless	t-stats	p-value (intergroup)
PD on MB at the sixth month	3.96 ± 0.57	3.43 ± 0.42	2.35	0.030*
PD on MB at the 12^th^ month	2.86 ± 0.35	2.66 ± 0.39	1.21	0.243
Paired t-test	11.67	7.87		
p-value (intragroup)	0.001*	0.001*		
PD on ML at the sixth month	3.79 ± 0.49	3.29 ± 0.32	2.69	0.015*
PD on ML at the 12^th^ month	2.84 ± 0.43	2.60 ± 0.41	1.28	0.218
Paired t-test	8.82	8.85		
p-value (intragroup)	0.001*	0.001*		
PD on DB at the sixth month	3.86 ± 0.58	3.21 ± 0.53	2.61	0.018*
PD on DB at the 12^th ^month	2.81 ± 0.43	2.54 ± 0.39	1.46	0.163
Paired t-test	10.25	6.93		
p-value (intragroup)	0.001*	0.001*		
PD on DL at the sixth month	3.72 ± 0.51	3.17 ± 0.48	2.47	0.024*
PD on DL at the 12^th^ month	2.84 ± 0.34	2.62 ± 0.41	1.31	0.211
Paired t-test	7.76	4.25		
p-value (intragroup)	0.001*	0.002*		

Comparison of the CBH at different time intervals between the flap and flapless implant placement groups showed a gradual reduction in both groups over time. At baseline, the mean CBH height at the mesial site was 12.37 ± 1.61 mm in the flap group and 12.19 ± 1.69 mm in the flapless group (p = 0.513), with similar values at the distal site (p = 0.611). By the sixth and 12th months, the CBH had slightly decreased in both groups, but there were no statistically significant differences between them at any time point (p > 0.05). The Friedman test showed a significant reduction in CBH over time in each group (p = 0.001). These findings indicate that both surgical approaches led to a similar pattern of crestal bone loss, suggesting that flapless implant placement does not significantly reduce bone resorption compared to the flap approach (Table 5).

**Table 5:** Intergroup comparison using the Mann-Whitney U test and intragroup comparison using the Friedman test for the crestal bone height (CBH) in mm at various time intervals. *p-value < 0.05: significant. Data is presented in the form of mean and standard deviation (SD).

Time interval	Flap	Flapless		U-stat	p-value (intergroup)
CBH on mesial at day 1	12.37 ± 1.61	12.19 ± 1.69		0.65	0.513
CBH on mesial at the sixthday	11.92 ± 1.61	11.82 ± 1.70		0.12	0.908
CBH on mesial at the 12^th^ day	11.58 ± 1.58	11.57 ± 1.74		0.34	0.731
Friedman test	20	20			
p-value (intragroup)	0.001*	0.001*			
CBH on distal at day 1	12.37 ± 1.56	12.2 ± 1.67		0.51	0.611
CBH on distal at the sixthday	11.94 ± 1.59	11.85 ± 1.69		0.23	0.819
CBH on distal at the 12^th ^day	11.49 ± 1.65	11.58 ± 1.66		0.65	0.518
Friedman test	20	20			
p-value (intragroup)	0.001*	0.001*			

## Discussion

This study aimed to compare the clinical outcomes between flap and flapless implant placement techniques, such as postoperative pain, PD, and CBH, over a 12-month follow-up period post functional loading. These findings highlight several key differences between the two techniques, with implications for clinical practice.

The VAS has been considered a reliable tool for pain assessment [12]. The results of our study indicated a significant difference in the postoperative pain levels between the two cohorts. Patients who underwent flapless implant placement exhibited markedly lower pain scores on both the third and seventh postoperative days than those who received implants via the flap technique. On the third day, the mean VAS score in the flap group was nearly twice that of the flapless group, thereby underscoring the less invasive nature of the flapless surgical procedures. This discrepancy persisted on the seventh day, albeit with a reduction in pain levels across both groups over time. These results align with the prevailing literature, which posits that flapless surgery mitigates postoperative discomfort attributable to reduced soft tissue manipulation and preservation of vascular integrity [5,13,14]. By circumventing flap elevation, periosteal blood vessels remain undisturbed, which may contribute to diminished inflammation and expedited tissue recovery.

In the realm of clinical decision-making, the management of postoperative pain constitutes a pivotal factor, particularly when viewed through patient-centered care. Alleviation of pain associated with flapless surgical techniques has the potential to augment patient satisfaction and enhance compliance with postoperative care regimens. Furthermore, a more expedited resolution of pain may facilitate a swifter return to routine activities for patients, thereby further accentuating the advantages of the flapless methodology with respect to postsurgical recovery [15].

PD measurements at diverse peri-implant locations exhibited statistically significant variations between the two cohorts, particularly at the six-month assessment. The flap group presented elevated PD values across all evaluated sites compared to the flapless group. This observation implies that flapless implant insertion may confer a more advantageous effect on soft tissue healing during the initial stages of osseointegration. A reduced PD is generally indicative of healthier peri-implant soft tissue conditions and a diminished likelihood of peri-implantitis. Maintenance of soft tissue architecture during flapless procedures could elucidate this benefit. As the flapless approach reduces surgical trauma to adjacent gingival structures, the biological width and integrity of the mucosal seal are likely to be preserved more effectively, resulting in less crestal bone resorption and shallower peri-implant sulci. In a systematic review by Gao et al. [16], 14 randomized control trials were included, and it was reported that VAS scores were significantly lower in the flapless group after 24 h; however, after the third day, this difference was reduced. Similarly, the flapless group showed significantly lower PD.

Interestingly, at the 12-month follow-up, the PD values decreased in both groups and the differences between them were no longer statistically significant. This finding suggests that while the flapless approach may confer early soft tissue healing benefits, the long-term soft tissue outcomes appear to be equal between the two techniques. This is consistent with a previous study reporting that soft tissue healing and maturation around implants tend to stabilize over time, regardless of the surgical approach used [17]. The augmented PD could potentially be attributed to the more pronounced inflammatory infiltration and fibroplastic response surrounding the incisions in the flap cohort [18].

Evaluation of the CBH indicated no notable discrepancies between the groups at any assessed interval. Both the flap and flapless cohorts exhibited a progressive and statistically significant decline in the CBH throughout the 12-month follow-up period. The trajectory of bone loss was analogous, suggesting that flapless surgical approaches do not provide a meaningful benefit in mitigating crestal bone resorption compared to traditional flap methodologies. This finding aligns with a systematic review by Gao et al. [16], who reported comparable bone-level changes between the two approaches. Although it has been hypothesized that flapless implant placement might limit bone remodeling by preserving the periosteal blood supply, the present data suggest that other factors, such as implant design, loading protocols, and patient-related variables, may play a more significant role in determining long-term crestal bone stability [17].

It is crucial to acknowledge the multifaceted characteristics associated with crestal bone remodelling. Although surgical methodology plays a significant role in the initial healing processes, the sustained integrity of bone is also determined by various elements, including the placement of implants, occlusal forces, oral hygiene practices, and systemic health parameters [19]. The analogous outcomes of the CBH observed in this investigation may imply that both methodologies, when executed appropriately, offer a conducive environment for the preservation of the bone surrounding dental implants.

Although a statistically significant difference was observed between the two groups in PD at six months, the clinical relevance of this difference remains minimal, as the values in both groups consistently fell within the accepted normal physiological range. This indicates that the observed differences may not have substantial implications for patient outcomes. Furthermore, over the long term, this difference notably diminished, particularly in the flap group, suggesting a process of natural soft and hard tissue remodeling and adaptation. This highlights that while the surgical technique may influence early healing outcomes, both approaches ultimately support stable peri-implant conditions.

Clinical implications

The notable decrease in postoperative discomfort and the advantageous early soft tissue recovery associated with flapless implant placement may render it an appealing option for both practitioners and patients. Nevertheless, it is crucial to emphasize that flapless methodology necessitates meticulous case selection and surgical proficiency. Insufficient visualization during flapless procedures could increase the risk of incorrect implant placement or harm to surrounding anatomical structures, particularly in individuals with restricted bone volume or intricate anatomical factors [20].

Furthermore, although flapless surgical approaches present numerous clinical benefits, they may not be applicable in all scenarios. For instance, in cases that require bone augmentation or in individuals with diminished bone quality, flap elevation may still be imperative to facilitate access for grafting interventions or ensure optimal implant positioning. Consequently, decisions regarding the utilization of flap versus flapless methodologies must be tailored to the individual, considering patient-specific anatomical and clinical variables, in addition to the surgeon’s level of expertise.

Limitations of the study

This investigation has numerous constraints. First, the relatively modest sample size of 20 patients constrains the applicability of the results and diminishes the statistical strength of identifying subtle variations between cohorts. Moreover, the non-randomized framework, wherein group assignment was determined based on clinical discretion and patient choice, introduces the possibility of selection bias, which may affect the outcomes. The duration of follow-up was restricted to 12 months post-functional loading, which may not adequately reflect enduring disparities in peri-implant bone stability or soft tissue integrity between the flap and flapless methodologies. Additionally, although the study utilized standardized radiographic approaches, slight discrepancies in angulation or stent alignment could influence the precision of the CBH evaluations. Pain evaluation was based on self-reported VAS scores, which are inherently subjective and susceptible to fluctuations based on individual pain tolerance. All surgical interventions were conducted at a single center, potentially restricting the external validity of the findings across diverse clinical environments or among practitioners with varying degrees of proficiency. Several factors may have influenced the outcomes of this study. Sex differences and implant location (maxillary vs. mandibular) were not controlled or stratified for, which could affect pain perception, healing rates, and crestal bone changes. Future studies with larger sample sizes and subgroup analyses could explore these potential confounders. Additionally, the probe depth measurement technique was standardized around the functional restoration rather than the abutment, which may have contributed to variability in assessment. Finally, radiographic limitations included the use of 2D periapical radiographs, which may not accurately detect changes in peri-implant alveolar bone due to superimposition of adjacent structures, and the lack of more advanced imaging techniques like CBCT may have limited the precision of our measurements.

## Conclusions

In summary, this study indicates that flapless implant insertion is linked to markedly diminished postoperative pain and lower initial peri-implant PD values at six-month post-functional loading with no statistically significant differences at 12-month post-functional loading, when compared with the flap technique, without adversely affecting long-term CBH. These results imply that in suitably chosen scenarios, flapless implant placement can improve initial patient comfort and facilitate soft tissue healing while preserving similar bone-level results. Subsequent investigations with larger cohorts and extended follow-up durations may yield additional understanding of the long-term clinical efficacy and patient-reported results associated with these two surgical methodologies.
